# Lower tumorigenesis without life extension in rats receiving lifelong deep ocean minerals

**DOI:** 10.1002/cam4.3028

**Published:** 2020-04-03

**Authors:** Suchada Saovieng, Jinfu Wu, Wei‐Horng Jean, Chih‐Yang Huang, Matthew F. Higgins, Ahmad Alkhatib, Mallikarjuna Korivi, Chiao‐Nan Chen, Chia‐Hua Kuo

**Affiliations:** ^1^ Laboratory of Exercise Biochemistry University of Taipei Taipei Taiwan ROC; ^2^ College of Sports Science & Technology Mahidol University Salaya Thailand; ^3^ Laboratory of Regenerative Medicine in Sports Science School of Physical Education & Sports Science South China Normal University Guangzhou City China; ^4^ Department of Anesthesiology Far East Memorial Hospital New Taipei Taiwan ROC; ^5^ College of Medicine Hualien Tzu Chi Hospital Tzu Chi Medical Foundation Tzu Chi University Hualien Taiwan ROC; ^6^ Graduate Institute of Biomedical Sciences China Medical University Taichung Taiwan ROC; ^7^ Cardiovascular and Mitochondrial Related Disease Research Center Hualien Tzu Chi Hospital Buddhist Tzu Chi Medical Foundation Hualien Taiwan ROC; ^8^ Center of General Education Buddhist Tzu Chi Medical Foundation Tzu Chi University of Science and Technology Hualien Taiwan ROC; ^9^ Department of Medical Research China Medical University Hospital China Medical University Taichung Taiwan ROC; ^10^ Human Sciences Research Centre University of Derby Derby UK; ^11^ School of Health and Life Sciences Teesside University Middlesbrough UK; ^12^ College of Physical Education Zhejiang Normal University Jinhua Zhejiang P.R. China; ^13^ Department of Physical Therapy and Assistive Technology National Yang Ming University Taipei Taiwan ROC

**Keywords:** cancer, fructose, glutathione, lifespan, tumor

## Abstract

Naturally occurring tumor in animals receiving high minerals from deep oceans (DOM: hardness 600 mg/L) from 6 months of age until natural death was firstly assessed in 200 Sprague Dawley rats, randomized into four groups: Control (C), DOM (D), Fructose (F), and Fructose + DOM (FD). Fructose drink contained 11% fructose. Tumor incidence (necropsy at death) in the D group was ~40% lower than that in the C group (*P* < .05), together with lower body mass gain and greater locomotive activity during their initial 18 months (*P* < .05) but not during later life. X‐ray image analysis on abnormal solid tissue among survivors at 18 and 24 months of age confirms a similar trend, exhibiting ~50% and ~65% lower tumor incidence than the C and F groups, respectively. Reduced‐to‐oxidized glutathione ratio (GSH/GSSG) declined with age for the first three quarters of life on all groups (*P* < .05), followed by a resurgence during end‐life among survivors at 24 months. This resurgence is markedly associated with lower tumor expansion but unrelated with DOM supplementation. Our results demonstrate valuable application of minerals and trace elements from deep oceans, as a vastly available natural source, on tumor suppression during normal aging.

## Graphical abstract

Tumor incidence increases with advancing aging. Lifelong supplementation of deep ocean minerals significantly suppresses age‐associated tumorigenesis. This tumor‐suppressive action is associated with preventing weight gain and increasing spontaneous physical activity during the first two‐thirds period of life.

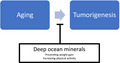

## 1 INTRODUCTION

Deep oceans have been proposed as a possible site for the origins of life.^1^ If terrestrial organisms are survived descendants who evolved from deep oceans, supplementation of deep ocean components to terrestrial animals should replenish loss of nutritive complexity associated with evolutionary sea‐to‐land migration. Deep oceans contain a great variety of minerals and trace elements, supporting a massive amount of marine organisms.^2^ However, ocean surface water is exhausted of biogenic nutrients, to some extent, by photosynthesis in the light permeable zone (~200 m). This is supported by superior health benefits of deep ocean water for terrestrial mammals than surface water with a similar major mineral profile.^3^

Studies in cell culture have suggested an inhibitory effect of DOM on the metastatic potential of breast cancer cells^4^ and colorectal adenocarcinomas cells.^5^ While most of in vivo studies are documented using tumor‐implanted young mammals, tumor incidence is generally known to increase during late life. Tumor incidence is partly associated with a lack of minerals^6^, ^7^ and seems to involve with an age‐dependent decline in GSH/GSSG levels.^8^, ^9^ However, a longitudinal investigation on GSH/GSSG changes over the lifespan, together with tumor incidence, has not previously been reported in mammals supplemented with a high concentration of minerals and trace elements.

The life‐prolonging effect of DOM consumption in mammals has been previously reported under pathogenic conditions.^10^, ^11^ Approximately 57% of the ovariectomized mice survived up to 16.6 months of age with DOM supplementation, whereas the untreated mice died at around 10 months of age.^11^ For streptozotocin‐induced diabetic rats, the survival rate was ~3 times higher in rats consuming DOM with moderate improvement in fasting glucose levels compared with the control rats.^10^ Tumor incidence during aging was not reported in both studies.

It is currently unknown whether prolonged consumption of desalinated DOM, containing high concentration of minerals and trace element, influences tumor incidence, survival time and physical vitality of naturally aging terrestrial animals. Therefore, the current study examines whether tumor incidence, longevity, and physical vitality in naturally aging rats can be altered by lifelong DOM drinking.

This study hypothesizes that components in DOM are able to compensate the loss of molecular complexity for terrestrial mammals associated with evolutionary sea to land migration. To evaluate this concept, survival time, tumor incidence, and physical vitality were measured.

Since fructose is a commonly used sweetener in beverages and is known to promote tumor growth,^12^ DOM drink was delivered in both fructose and fructose‐free forms.

## 2 METHODS

### 2.1 Animals

A total of 200 Sprague Dawley rats (sex balanced) with known date of birth were obtained from LASCO Corporation. Rats were transferred to the Animal Center of University of Taipei at 1 month of age (weighed 111 ± 1 g). Two animals were housed per cage and provided standard laboratory chow (LabDiet). Cages were kept in an animal room with 12/12 hours light/dark cycle, 22 ± 2°C, and 50% relative humidity.

### 2.2 Experimental design

To evaluate the effect of DOM on aging‐associated tumorigenesis, vitality and longevity, the six rats that survived less than 12 months were excluded from analysis. Rats were randomized into the following four groups: Control (C, n = 49), DOM (D, n = 48), Fructose (F, n = 49), Fructose + DOM (FD, n = 48). A DOM drink with or without fructose (11% W/V) was provided daily from 6 months of age until natural death. Tap water used to dissolve fructose and DOM was served as the control drink. Food and drink were provided ad libitum for all animals, with consumption volume recorded every 3 days. Total energy intake from standard chow was calculated according to nutritional information provided by the manufacturers (Normal Chow #5001, LabDiet) and where applicable added to the fructose consumption in the drink. Body composition, spontaneous locomotive activity, fasting glucose, glucose levels during insulin tolerance test (ITT), reduced glutathione (GSH) to oxidized glutathione (GSSG) ratio, and tumor size progression (X‐ray image) were measured at the age of 6th, 12th, 18th, and 24th months.

### 2.3 Deep ocean minerals

The DOM used in this study was supplied by Taiwan Yes Deep Ocean Water Co., LTD, collected from the depth of 662 meters below earth's surface in the West Pacific Ocean, Hualien, Taiwan. The DOM drink was diluted in tap water from a concentrate of desalinated seawater with or without fructose (11%) to reach a hardness of 600 mg/L, according to a previous study.^13^ Table 1 shows the mineral and trace element profile of the DOM used in this study. Consistency of major mineral levels of the DOM in the past 5 years has been confirmed for potassium, sodium, magnesium, chloride, bromide, and calcium content under the same hardness (pH 8.1).

**TABLE 1:** Mineral and trace element profile of the deep ocean‐based mineral water

Mineral	DOM (mg/L)	Control (mg/L)
Ca	15	14
Mg	165	0.8
K	38	0.3
Na	109	10

Trace element	DOM (µg/L)	Control (µg/L)
Li	44	24
Rb	64	50
B	4725	100

### 2.4 Blood sample collection

Blood samples were always collected after 12 hours of fasting from tails of rats into an EDTA containing tube. After centrifugation of blood samples at 3000 rpm for 10 minutes at 4°C, plasma samples were transferred into Eppendorf tubes for storage at −80°C until analysis.

### 2.5 Reduced and oxidized glutathione

Glutathione fluorometric assay kits (Biovision) were used to measure reduced glutathione (GSH) and oxidized glutathione (GSSG) levels. Briefly, 60 μL of plasma samples were centrifuged in a tube containing 20 μL ice‐cold perchloric acid, vortexed, and kept on ice for 5 minutes, then spun for 2 minutes at 13 000 g at 4°C and the resultant supernatant collected. The supernatants were then assayed according to the manufacturer’s instructions, using an ELISA reader (Tecan GENios, A‐5082). Glutathione levels were expressed as μg/mL.

### 2.6 Insulin tolerance test (ITT)

After 12 hours of fasting, anesthetized rats received an intraperitoneal insulin injection (0.3 U/kg body mass). Blood glucose levels (mg/dL) were measured immediately using the Accu‐chek® performa system (Roche Diagnostics) before (0 minutes) and 30, 60, 90, and 120 minutes after insulin injection. Plasma insulin levels (μg/L) were measured on the same day of blood collection using an enzyme‐linked immunosorbent assay (ELISA) kit (Mercodia, Mercodia AB). Optical density values were read at 450 nm using an ELISA reader (Tecan GENios, A‐5082).

### 2.7 Dual‐energy X‐ray absorptiometry (DEXA)

Body composition of rats was measured by DEXA (Lunar iDXA, GE Medical Systems) and analyzed using a specific small animal software package. Body composition was determined by image analysis using GE's Lunar software enCORE^™^ (GE Medical Systems). DEXA scans were performed under isofluorane anesthesia following 12 hours of fasting. Body composition data including lean mass (g), fat mass (g), bone mass (g), bone mineral density (BMD) (g/cm^2^), and % bone mass (% body mass) were recorded.

### 2.8 Tumor assessments

Necropsy after natural death was used for tumor assessment to generate data on tumor incidence. For necropsy analysis, deceased rats had gross autopsies by a certified specialist. To assess tumor size expansion, abnormal solid tissues on X‐ray image from DEXA were measured at 6th, 12th, 18th, and 24th months. Tumor volume (V) was determined by the largest (a) and smallest (b) superficial visible diameters on x‐ray images and calculated based on formula [*V* = *a* × (*b* ^2)^/2].^14^

### 2.9 Spontaneous locomotive activity

To assess physical vitality of the animals, spontaneous locomotor activity was monitored using a Locoscan video tracking system (Clever Sys, VA, USA) on a black plastic cage (40 cm × 40 cm).^15^ One week prior to assessment, rats were familiarized to the plastic cage for 10 min/day with spontaneous locomotive activity measured in quiet and dark environments. Rats were placed in the center of the plastic cage and allowed to move freely. Daily spontaneous locomotive activity of rats was recorded for 20 minutes by the camera mounted above the center of the testing cage. The first and last 5 minute periods were removed from analysis to avoid disturbances related to human access to the dark room. Artifact due to feces and urine was removed. Ten‐min video data were analyzed using the Locoscan software (Clever Sys) providing values on horizontal movement distance (cm) and movement speed (mm/s).

### 2.10 Statistical analysis

Survival curves for different groups were presented by Kaplan‐Meier curve. One‐way analysis of variance (ANOVA) was conducted to determine the difference among four groups of all variables. Repeated measure analysis was used to determine the group difference of all variables within the same survivors at different time points (6th, 12th, 18th, 24th month) for 24th month. The Duncan *post hoc* test was used to distinguish mean differences between pair of groups. A two‐way ANOVA was used to analyze the treatment effects of the DOM and fructose drinks, and time effects over the animal lifespan. The difference in tumor incidence among groups was compared by Chi‐squared test. All values are expressed as mean ± standard error (SE). A level of *P* < .05 was set for statistical significance for all tests.

## 3 RESULTS

### 3.1 Lifespan

**FIGURE 1:**
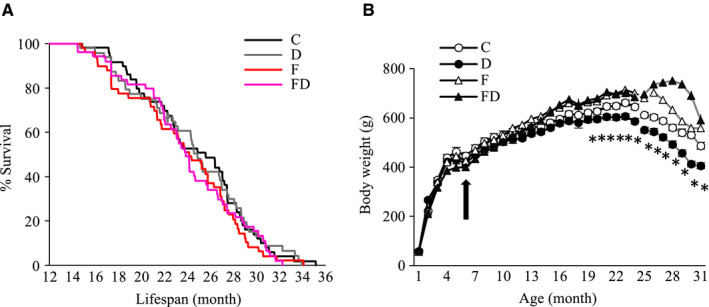
Longevity and body mass. No significant difference in cumulative survival (A) of the rats (n = 194, average lifespan: ~24 mo) survived over 12 mo among 4 groups (C, n = 49; D, n = 48; F, n = 49; FD, n = 48). Trajectory lines represent evolution of mean body mass of survivors (n = 102) at 24 mo (B) (C, n = 26; D, n = 28; F, n = 25; FD, n = 23). Significant body mass loss was observed for all groups after 24 mo of age. The D group had the lowest body mass from 19 mo of age. *P < .05 compared with C, F, and FD groups. Abbreviation: C, Control; D, Deep ocean mineral water; F, Fructose; FD, Fructose + Deep ocean mineral water

Data for longevity and body mass trajectory are shown in Figure 1. The average lifespan of each group was ~24 months (Figure 1A). No significant difference in average lifespan was observed among groups (C: 749 ± 25 days; D: 725 ± 23 days; F: 709 ± 25 days; FD; 717 ± 21 days). For body mass trajectory (Figure 1B), only those rats survived at 24 months were included to calculate the mean mass (n = 102). Body mass in the fructose‐fed groups was significantly greater than that in the non‐fructose‐fed groups (*P* < .05) overtime. Body mass of the D group was significantly lower than that of the C group during the same period (*P* < .05). Survivor numbers declined rapidly after 24 months of age for all groups (Figure 1B). Rapid loss of body mass before death is a common feature during end stage of life for all groups (after 24 months of age, body mass values are averaged among survivors). Rats in the fructose‐fed rats had more body mass than those in the fructose free group among survivors after 24 months of age (main treatment effect, *P* < .05). D group showed lowest body mass compared with the rest of other groups during the same period (*P* < .05 vs C group).

### 3.2 Food and water consumption

**FIGURE 2:**
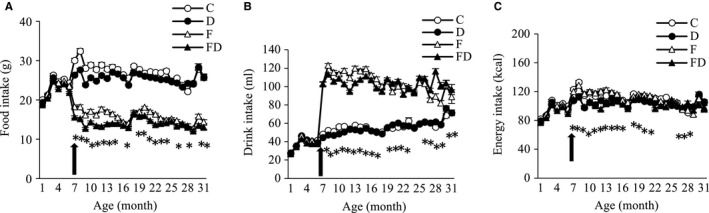
Food and drink consumption. Fructose drinks reduced food consumption by nearly 50% (A) and substantially increased water consumption (B) from 6 mo of age. Total energy intake in the DOM‐treated groups was moderately lower than those in the non‐DOM groups from 6 to 12 mo of age (*P* < .05) (C). Trajectory lines represent mean values of each group (n = 102) survived at 24 mo (B) (C, n = 26; D, n = 28; F, n = 25; FD, n = 23). Abbreviation: C, Control; D, Deep ocean mineral water; F, Fructose; FD, Fructose + Deep ocean mineral water. The arrow in the figure represents the time when the experiment was started

Fructose and DOM interventions started from 6 months of age. Food, water, and total energy intake from rats survived at 24 months are shown in Figure 2. Food consumption decreased (Figure 2A) and water consumption (Figure 2B) increased significantly in the fructose‐fed groups after 6 months of age. Energy distribution in fructose‐fed groups was ~40% derived from fructose. Food, water, and energy intake (Figure 2C) in the DOM groups were lower than those in the DOM‐free groups after the DOM intervention at 6 months of age (main treatment effect, *P* < .05).

### 3.3 Tumor incidence

**FIGURE 3:**
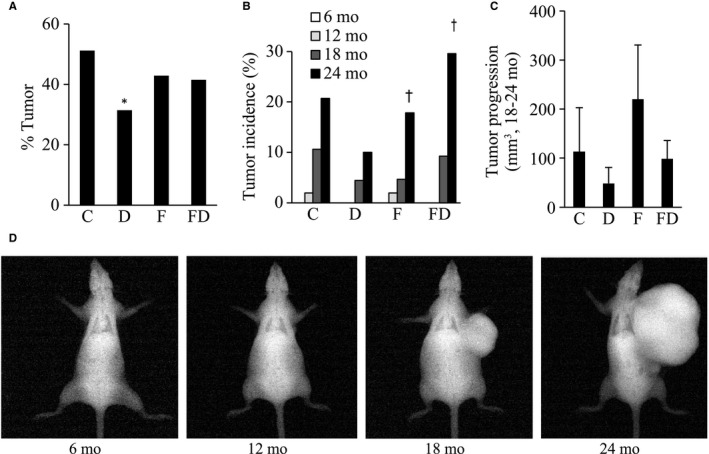
Tumor incidence and tumor size expansion. Tumor incidence (necropsy) of the rats in the D group was ~40% less than that in the C group (A). Representative X‐ray tumor image of a rat (D), measured every 6 mo until 24 mo of age. Age‐dependent tumor incidence (X‐ray image) increased dramatically from 18 to 20 mo of age, in which fructose‐fed groups showed greater increases in tumor incidence than those non‐fructose‐fed groups (B). Tumor size expansion from 18 to 24 mo showed similar trend among four groups but no statistical significance is reached (C). Abbreviation: C, Control; D, Deep ocean mineral water; F, Fructose; FD, Fructose + Deep ocean mineral water. *P < .05 compared against the C group; ^†^ *P* < .05 compared between 18 and 24 mo

Tumor incidence, examined by necropsies at death (Figure 3A), of the D group was ~40% less than that of the C group (*P* < .05). Tumor size development, based on abnormal solid tissue on X‐ray image (Figure 3D), was measured every 6 months until 24 months of age. Tumor incidence in the fructose‐fed survivors was significantly increased between 18 and 24 months (Figure 3B; main time effect, *P* < .05). Tumor size expansion from 18 to 24 months of age showed similar trend with aforementioned tumor incidence but statistical significance is not reached (*P* = .24 vs C group; *P* = .07 vs F group; *P* = .16 vs FD group) (Figure 3C). Tumor location and number among four treatment groups are detailed in Table 2.

**TABLE 2:** Location and number of spontaneous tumors among 68 (26 males and 42 females) tumor‐bearing rats

Location	Number of tumors				Total
	Groups				
	C	D	F	FD	
Male					
Subcutaneous	1		2	2	5
Extraperitoneal	1	1			2
Prostate	1	1	1		3
Lung	6		1	4	11
Liver	2	1		1	4
Kidney			2		2
Gallbladder	1				1
Female					
Subcutaneous	8	10	10	12	40
Ventral	2		3	1	6
Extraperitoneal	3			1	4
Intraperitoneal			1		1
Ovary		2	1	1	4
Lung	1		3	3	7
Liver		1			1
Adrenal gland			2		2
Nasal cavity			1		1
Uterus				1	1
Total	**26**	**16**	**27**	**26**	**95**

### 3.4 Blood glucose and insulin

**FIGURE 4:**
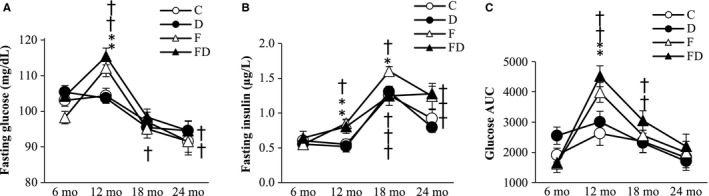
Glucose and insulin. No significant effect of DOM treatment on fasting glucose (A), fasting insulin (B), and glucose AUC (C) in blood of the survivors (n = 102) at 24 mo (C, n = 26; D, n = 28; F, n = 25; FD, n = 23) are detected according to two‐way ANOVA. Significant fructose effect on fasting glucose and glucose AUC was observed at 12 mo of age, but this effect was diminished at 24 mo of age. Abbreviation: C, Control; D, Deep ocean mineral water; F, Fructose; FD, Fructose + Deep ocean mineral water, Glucose area under curve (Glucose AUC). *P < .05 compared against the C group at the same time point; ^†^ *P* < .05 compared against their baseline value at 6 mo

Trajectories of fasting glucose, fasting insulin, and glucose AUC of the rats survived at 24 months are shown in Figure 4. Fasting glucose (Figure 4A) and glucose AUC (Figure 4C) in the fructose‐fed rats were increased at 12 months and gradually declined thereafter. Plasma insulin levels (Figure 4B) in the rats from all groups increased to its peak at 18 months of age and declined thereafter by 22% until 24 months of age (main time effect, *P* < .05). Insulin levels in the fructose‐fed rats were significantly greater than those in the fructose‐free rats at 12 months of age (main treatment effect, *P* < .05). No significant differences in fasting glucose, insulin, and glucose AUC between the DOM‐treated and DOM‐free groups were observed.

### 3.5 Body composition

**FIGURE 5:**
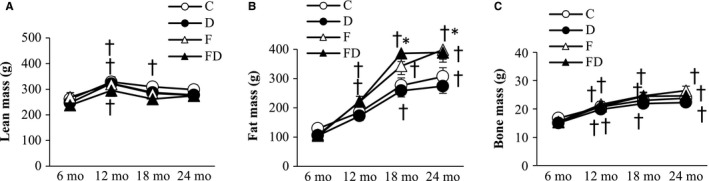
Body composition. Lean mass slightly increased from 6 to 12 mo of age and decreased thereafter (A). Fat mass increased progressively from 6 mo and reaching plateau after 18 mo of age (B). Significant fructose effect on fat mass was observed from 18 to 24 mo of age (main effect). These data were collected based on the survivors (n = 102) at 24 mo (C, n = 26; D, n = 28; F, n = 25; FD, n = 23). Bone mass showed similar trend as fat mass, but fructose effect was less notable (C). Abbreviation: C, Control; D, Deep ocean mineral water; F, Fructose; FD, Fructose + Deep ocean mineral water dual‐energy x‐ray absorptiometry. *P < .05 compared against the C group at the same time point; ^†^ *P* < .05 compared against their baseline value at 6 mo

Trajectories of lean mass and fat mass of rats survived at 24 months are shown in Figure 5. Lean mass increased significantly from 6 to 12 months and remained stable until 24 months of age. No treatment effects of DOM and fructose were found on lean mass (Figure 5A). From 6 to 24 months of age, fat mass (Figure 5B) from all groups increased progressively (main time effect, *P* < .05). Fat mass in the fructose‐fed rats was significantly greater than those in the non‐fructose‐fed rats (main treatment effect, *P* < .05), especially from 12 to 24 months (*P* < .05). Bone mass increased (Figure 5C), whereas % bone mass decreased with age from 12 to 24 months of age (main time effect, *P* < .05). No treatment effect of DOM was found on bone mass, BMD, and bone mass.

### 3.6 Physical vitality

**FIGURE 6:**
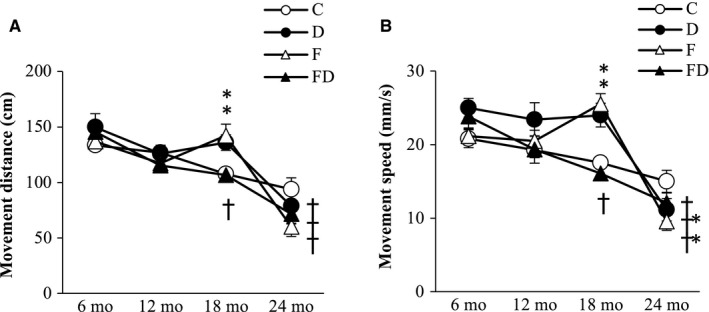
Physical vitality. Horizontal movement distance (A) and movement speed (B) decreased with age. However, the D and F groups sustained higher spontaneous locomotive activity until 18 mo of age. The trajectory data were collected based on the survivors (n = 102) at 24 mo (C, n = 26; D, n = 28; F, n = 25; FD, n = 23). Abbreviation: C, Control; D, Deep ocean mineral water; F, Fructose; FD, Fructose + Deep ocean mineral water. *P < .05 compared against the C group at the same time point; ^†^ *P* < .05 compared within the same group against the baseline at 6 mo

Horizontal movement distance (Figure 6A) and movement speed (Figure 6B) declined progressively from 6 to 24 months of age (main time effect, *P* < .05). This age‐dependent decline was delayed in the D and F groups until 18 months of age (*P* < .05 vs C group). At 24 months of age, both locomotive measurements were similar among groups.

### 3.7 Plasma redox status

**FIGURE 7:**
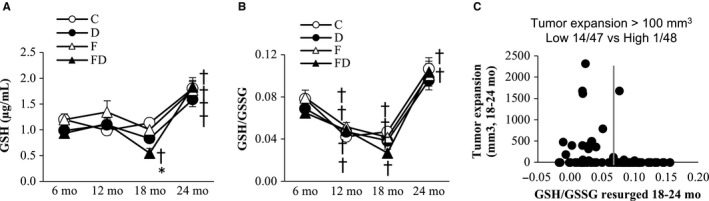
Plasma glutathione. Reduced glutathione (GSH) (A) and reduced‐to‐oxidized glutathione ratio (GSH/GSSG) (B) decreased with age until 18 mo and resurged at 24 mo of age above 6 mo level. Scatter plot between tumor size progression and magnitude of GSH/GSSG resurged from 18 to 24 mo of rats (low GSH/GSSG resurged group vs high GSH/GSSG resurged group) (C). High GSH/GSSG resurged group showed low incidence of tumor progression. These data were collected based on the survivors (n = 102) at 24 mo (C, n = 26; D, n = 28; F, n = 25; FD, n = 23). Abbreviation: C, Control; D, Deep ocean mineral water; F, Fructose; FD, Fructose + Deep ocean mineral water. *P < .05 compared against the C group at the same time point; ^†^ *P* < .05 compared within the same group against the baseline at 6 mo

Trajectories of redox state (GSH/GSSG ratio) of rat survivors at 24 months are shown in Figure 7. The plasma levels of GSH (Figure 7A) and GSH/GSSG ratio (Figure 7B) decreased progressively with age (main time effect, *P* < .05), until 18 months and resurged at 24 months of age by 61% and 40% respectively above their 6‐month levels (*P* < .05). Plasma GSSG increased by ~50% with age from 6 to 12 months and remained stable until 24 months of age. No difference in GSH/GSSG ratio was found among groups. When all groups were combined and evenly divided into two groups according to the change of GSH/GSSG ratio from 18 to 24 months of age, rats on the lower halves of GSH/GSSG ratio increase showed substantially greater tumor progression (14/47 cases with tumor size expansion >100 mm^3^) compared with their higher halve counterparts with only 1 out of 48 cases showing tumor size expansion >100 mm^3^ (Figure 7C).

## 4 DISCUSSION

This study hypothesized that components in DOM are able to compensate for the loss of molecular complexity for terrestrial mammals associated with evolutionary sea to land migration. To evaluate this concept, survival time, tumor incidence, and physical vitality were measured. The main findings of the study suggest the following: (a) Consuming DOM decreases tumorigenesis during mammalian aging. This benefit is offset by adding fructose into the drink; (b) DOM is neither toxic nor life‐prolonging for terrestrial mammals; (c) Daily low concentration fructose drinking does not significantly decrease longevity albeit increased fat gain and hyperinsulinemia were observed during early age; (d) An age‐dependent decline in the GSH/GSSG ratio occurs during the first 3 quarters of life followed by a resurgence at the end period of life regardless of treatments. This resurgence seems to be associated with an intrinsic compensatory mechanism against tumor formation during the end stages of life but appears unrelated with any DOM effect on tumor suppression.

The most noteworthy finding of the study is reduced tumor incidence in rats consuming DOM under fructose‐free condition versus all other groups. The underlying mechanism to explain this tumor‐suppression effect could be mediated by direct molecular interaction between chemical components of DOM and emerging tumor cells. Decreased tumor proliferation and metastasis in human breast cancer cell lines^4^ and HT‐29 colorectal cancer cells^5^ in media containing DOM have been previously known. DOM has enriched divalent ions such as magnesium. In humans, higher tumor incidence was observed among middle‐aged individuals (50‐76 years) with insufficient magnesium consumption (below RDA) in a 8‐year follow‐up study.^6^ In contrast, the role of trace elements in DOM on tumor incidence remains to be determined.

Another possibility to explain the lower tumor incidence with lifelong DOM supplementation is its suppressive effect on body mass development, secondary to suppressed energy intake. A direct relationship between tumorigenesis and body mass gain has been well established.^16^, ^17^, ^18^ Therefore, higher body mass in fructose‐free groups might explain why FD‐fed rats had not shown a lower tumor suppression. This possibility is also supported by our data, showing a substantially greater tumor incidence associated with quicker body mass gain in FD‐fed than fructose‐free rats (Figure 1). Slower body mass gain during growth over time in DOM‐treated rats is consistent with previous studies conducted in genetically obese mice and high‐fat diet (HFD)‐induced obese rats.^19^, ^20^ However, the effect of DOM on energy intake and body mass during early aging is moderate in our study.

We failed to find significant life‐prolonging effects of DOM consumption for these naturally aging mammals as reported by previous studies in ovariectomized senescence‐accelerated mice (OVX‐SAMP8) and streptozotocin‐induced diabetic rats.^10^, ^11^ This is probably associated with suppressed body mass gain after 19 months of age. Preventing body mass gain during the early phase of life is metabolically beneficial but loss of body mass at the late stage of life has been linked to increase mortality.^21^, ^22^

During the early period of life, age‐dependent body mass gain is a major cause of insulin resistance, hyperglycemia, tumorigenesis, and premature death for both animals and humans.^23^ Fructose is generally known to promote gains in body mass.^24^ It is thus intriguing that greater tumor incidence and hyperinsulinemia in rats consuming fructose drinks are not associated with shorter longevity. Our data shows that loss of body mass at the end of life is also attenuated by fructose. The loss of body mass at the end of life is closely associated with increased mortality for both rats and humans.^21^ Furthermore, the fructose concentration (11%) of the drink mimicking most of the sugar beverages used in humans was considerably lower compared with most of the previous studies showing negative metabolic survival outcomes.^25^, ^26^, ^27^ Compensatory hyperinsulinemia during body mass gain does not appear to be sustainable over time since insulin was reversed at the end of life according to the current results. A lower decline in insulin^28^ and body mass^29^, ^30^ during the end stage of the life in rats consuming fructose may explain why we failed to observe shorter longevity in the fructose‐fed rats.

The result of the study suggests that the GSH/GSSG resurgence is a physiological compensatory mechanism against age‐associated tumorigenesis. The GSH/GSSG ratio declines during aging.^31^ Very few studies have reported changes in GSH/GSSG until the end period of life. In the study, GSH/GSSG trajectories for the first three quarters of life (first 18 months) are consistent with existing knowledge. We observed a resurgence in GSH/GSSG at 24 months of age (~average lifespan) among survivors, which is consistent among all groups regardless of experimental treatments. Those animals showed substantially slower tumor expansion, which was independent of DOM consumption, with greater resurgence in GSH/GSSG at 24 months of age compared with their same age counterparts. Dietary GSH supplementation has been shown to suppress tumor formation induced by carcinogens in young animals^32^ or tumor progression induced by carcinogens in aging animals.^33^, ^34^ Depleting GSH increases tumorigenesis in colons fivefold in p53‐knockout mice compared with an untreated control group.^8^, ^9^ However, decreased tumor incidence in the DOM group does not appear to be related to the GSH/GSSG ratio.

## 5 CONCLUSION

Tumor suppression outcome with lifelong DOM consumption implicates an important role of minerals and trace elements in the deep ocean on naturally occurring tumorigenesis during mammalian aging. DOM are able to compensate loss of molecular complexity for terrestrial animals. Our data on longevity and physical vitality, suggest that DOM supplementation is safe but not life‐prolonging to normally aging terrestrial mammals.

## 6 ETHICS STATEMENT

This study was approved by the Animal Care and Use Committee at University of Taipei (approval number 20120006) and conducted in accordance to Taiwan's Animal Protection Act.

## CONFLICT OF INTEREST

CHK were invited as a scientific consultant for Taiwan Yes Corporation during the research period. Travel expenses for international scientific presentations and open access fee were supported by Taiwan Yes Corporation.

## AUTHORS’ CONTRIBUTIONS

SS, JW, and CHK had full access to all of the data in the study and took responsibility for the integrity of the data and the accuracy of the data analysis. SS and CHK carried out the study concept and design. SS and MK conducted intensive literature review during preparation of the manuscript. SS and CHK drafted the manuscript. SS, JW, WHJ, MFH, CYH, and AA conducted critical revision of the manuscript for important intellectual content. All authors have read and approved the final version of the manuscript and agreed with the order of presentation of the authors.

## Data Availability

Data will be made available upon request.
